# Correction: Pilot study assessing gut microbial diversity among sexual and gender minority young adults

**DOI:** 10.1371/journal.pone.0347379

**Published:** 2026-04-16

**Authors:** Ashley Guy, Shannon McAuliffe, Robbie Cross, Yue Zhang, Richard E. Kennedy, Norman R. Estes, Samantha Giordano-Mooga, Christine Loyd

Following publication of this article [1], the authors noted two errors.

Specifically:

- The published article reports samples sizes of CIS-HET = 43 and SGM = 22 for the microbiome analysis. These values correspond to the full enrolled sample, but only 41 participants returned stool samples. The correct sample sizes for the microbiome analysis are CIS-HET N = 27 and SGM N = 14. Subgroup sample sizes in the SGM group are corrected to 9 (sexual minority only) and 5 (gender and sexual minority).
- Table 1 did not include a p-value and sample size for Characteristic: Race.

The following text has been corrected:

The fifth sentence of the abstract is updated to: “Stool samples were returned by 41 participants and constituted the analytic sample for microbiome analyses (CIS‑HET N = 27; SGM N = 14).”

The Interviews and Gut microbial diversity sections of the Methods are updated.

Specifically:

- The first sentence of the Interviews section is updated to: After enrollment, all 65 participants (CIS‑HET N = 43; SGM N = 22) completed a 45-minute in-person interview, which occurred at the start of the semester.
- The third sentence of the interviews section is updated to: Additionally, participants were asked to collect a stool sample at home and return it to the research team for storage within 48hr following the interview. Trained assessors completed the interviews and collected biological samples.
- The fourth sentence of the first paragraph of the Gut microbial diversity section is updated to: Forty-one participants (CIS-HET N = 27; SGM N = 14) returned a stool sample for microbiome analysis. Participants that did not return samples within 48 hours of the interview were not included in the microbiome analysis. Of the 14 SGM samples, 9 were provided by sexual minority only participants while 5 were provided by gender and sexual minority participants.
- The second sentence of the second paragraph of the Results section is updated to: When sexual minorities only were analyzed separately from gender and sexual minorities compared to the CIS-HET group, alpha diversity was significantly reduced among sexual minorities only (N = 9; Shannon Alpha Diversity mean = 4.89, SD = 0.76) and gender and sexual minorities (N = 5; Shannon Alpha Diversity mean = 4.69, SD = 0.89) compared to the CIS-HET group.

Additionally, the authors have provided an updated Fig 1. See the correct figure and legend here.

Table 1 has been updated to include the p-value and sample size for Characteristic: Race. Please see the correct table here.

Please note that the concerns do not affect the results or conclusions for this study. A member of the Editorial Board reviewed the corrected Figure and Table and stated that the conclusions in [1] are still supported.

The authors apologize for the errors in the published article.

**Table 1:** Characteristics of the study sample.

Characteristic	All	CIS-HET	SGM	P Value	N
	*N = 65*	*N = 43*	*N = 22*		
Age, Mean (SD)	20.0 (1.66)	20.2 (1.70)	19.5 (1.50)	0.121	61
Race, No. (%)				0.375	63
African American/Black	18 (28.6)	12 (28.6)	6 (28.6)		
Asian/Asian India	6 (9.52)	6 (14.3)	0 (0.00)		
Caucasian	29 (46.0)	17 (40.5)	12 (57.1)		
Hispanic	9 (14.3)	6 (14.3)	3 (14.3)		
Middle Eastern/ North African	1 (1.59)	1 (2.38)	0 (0.00)		
PHQ-9 Score, Mean (SD)	8.18 (4.97)	8.09 (4.82)	8.36 (5.36)	0.843	65
GAD-7 Score, Mean (SD)	8.74 (5.41)	9.12 (5.29)	8.00 (5.70)	0.449	65
PSS-10 Score, Mean (SD)	18.3 (5.88)	17.8 (5.83)	19.1 (6.02)	0.427	65
Nail Cortisol (SD)	0.09 (0.23)	0.06 (0.15)	0.14 (0.33)	0.284	60
Saliva Cortisol (SD)	0.58 (0.59)	0.58 (0.55)	0.57 (0.69)	0.939	65
Shannon Alpha Diversity, Mean (SD)	5.17 (0.69)	5.35 (0.57)	4.81 (0.78)	0.034^*^	41

PHQ-9 = Patient Health Questionnaire 9, GAD-7 = Generalized Anxiety Disorder 7, PSS-10 = Perceived Stress Scale 10. Statistical significance *P < .05. Differences in variables between the cisgender heterosexual (CIS-HET) group and the sexual and gender minority (SGM) group are represented.

**Fig 1:**
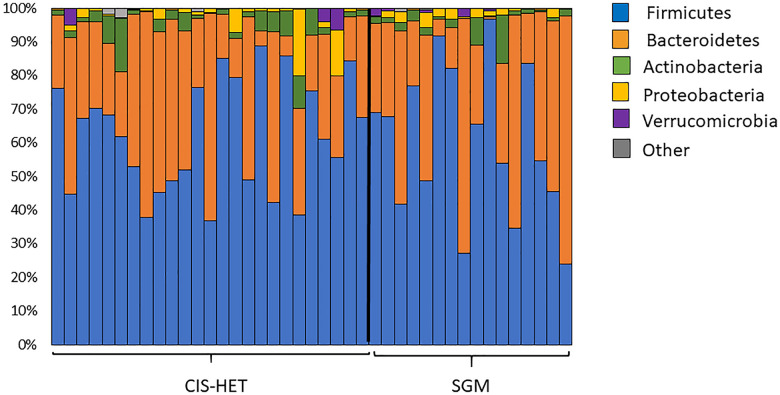
The average relative abundance of microbial phyla of the gut microbiome among the CIS-HET and SGM groups. The average relative abundance of major bacterial phyla in stool samples from cisgender-heterosexual (CIS-HET) and sexual and gender minority (SGM) participants. Bars represent individual participants (CIS-HET N = 27, SGM N = 14). Phyla composing <0.01% are combined and represented as other.
