# Supplementary material for: Pilot study assessing gut microbial diversity among sexual and gender minority young adults
Source: PLoS One. 2024 Jul 3;19(7):e0306638. doi: 10.1371/journal.pone.0306638 (PMC11221641; doi:10.1371/journal.pone.0306638)
Supplement: Attachment — Submitted filename: PLoS Review mar 2024.docx [file pone.0306638.s001.docx]

Thank you for the opportunity to review this important work. Gut microbiome research is crucially important, especially in the SGM population. I would have appreciated a great focus, including targeted recruitment, of the SGM population rather than using a cisgender group.

Please also address decision to combine sexual and gender minorities, as these groups are very different and have different risk factors for changes in gut microbiome that were not adequately addressed in this paper.

I look forward to seeing the revisions.

Abstract

The aim of this study is to compare gut microbiome in SGM and cis-het populations

This is a cross-sectional study, not prospective

Would say date range, not semesters (eg, 9/2021-5/2022)

Write out four, not 4-year

Unclear why the specific differences in alpha diversity would contribute to negative health effects among SGM community

Introduction

“expect” prejudice? Or experience prejudice? Would recommend explaining this a little bit

“comparatively”? compared to what?

Would not consider consuming a poor diet a physical or medical manifestation. That is a lifestyle component, not a health sequelae

Add hyphen “SGM-related” health disparities

This investigation aims to begin to understand is awkward and wordy. Please revise

Will help in beginning to develop a better understanding. Also awkward. Please revise

Methods

Inclusion criteria would also include age over age 18

Were participants compensated?

Seems like you could have done targeted recruitment of SGM populations since this was the group of interest. Was this done? Please comment

What was the decision to have a control group? Seems like a lot of study resources went to this group and perhaps existing data in the literature could have been used as a comparator since this is a pilot study

Screening data “were” collected—not was. Please change

Again would refrain from using “semesters” as this may be unclear to some readers. Would just use time points when relevant.

PSS-10 “has been shown” to be a valid measurement..not was. This implies that you did the validation

Sentence “PHQ-9…previously used among young adults to assess severity of depression.” Could just be included in second sentence of paragraph. Could say it has been validated…Major depressive disorder, including among young adults.

Was study powered enough to use the statistical tests referenced? Please comment

Discussion

The pilot aimed to compare microbial diversity of SGMs compared to cis-het

If you are going to write out sexual and gender minorities, then also write out cisgender heterosexual. Either that or don’t re-write either.

This is a pilot, be careful to say “SGM status is associated with disruptions”. Would say “may be” associated.

Would say health sequelae or negative health outcomes..rather than issues

Write out six species, not 6 species (less than ten, write out number)

When discussing the reasons for mental health outcomes, would include that these students are enrolled in college and may not be experiencing some of the typical SES challenges that cause minority stress in SGMs and that college campuses are typically very inclusive places.

Write out four (4-year university)

Include as limitation that this was only performed in a very geographically limited area

The decision to combine SGMs is problematic in that it is well-known that minority stress among sexual minorities differs greatly than gender minority stress. Also, GM can be on exogenous hormones which can change gut microbiome. These need to be addressed

“and the fact that it is something we can change” is poorly worded. Better to say that it is “modifiable”

Do not need to write out SGM again

SGM “may be associated” with reduction in alpha diversity…or specifiy that this occurred in this small pilot sample.

Would not say that programs for improving gut microbial diversyt could reduce health inequity. You have not shown that this difference is an inequity, especially since all the psychosocial factors were the same. Would just end sentence after “among SGMs.”
