# Supplementary material for: Pilot study assessing gut microbial diversity among sexual and gender minority young adults
Source: PLoS One. 2024 Jul 3;19(7):e0306638. doi: 10.1371/journal.pone.0306638 (PMC11221641; doi:10.1371/journal.pone.0306638)
Supplement: Attachment — Submitted filename: Response to Reviewers_2.docx [file pone.0306638.s003.docx]

May 28, 2024

**PLOS ONE**

**REF: Manuscript #:** PONE-D-24-05281

Dear Dr. Pallikkuth-

Thank you for your email of May 23, 2024. We have edited our manuscript and addressed the comment from reviewers below. We thank the reviewers for all their work on this manuscript and we hope this revision is satisfactory. We look forward to hearing from you again soon.

We have unpublished our raw data in figshare and will republish it making it public once again once it is accepted for publication. The DOI will remain the same: 10.6084/m9.figshare.25639596.

Reviewer comment:

(1) Presenting sexual and gender minorities as one group is still a concern. The authors need to satisfactorily address this comment
    a. ***Response***: We agree that examining differences between gender minorities and sexual minorities is important. We have completed additional analyses and determined that there was no difference between sexual minorities only and gender/sexual minorities (all gender minorities in this study also self-identified as sexual minorities) in terms of demographics and outcomes.

We have addressed this in the text.

**Line 195-197 in the results:** When the SGM group was separated into sexual minority only (N=14) and gender/sexual minorities (N=8), there was no significant difference between groups in demographics (age, race), psychological factors (PHQ-9, GAD-7, PSS-10), or nail and saliva cortisol levels measured. **Also, lines 217-220 in the results:** When sexual minorities only were analyzed separately from gender and sexual minorities compared to the CIS-HET group, alpha diversity was significantly reduced among sexual minorities only (N=14; Shannon Alpha Diversity mean=4.89, SD=0.76) and gender and sexual minorities (N=8; Shannon Alpha Diversity mean=4.69, SD=0.89) compared to the CIS-HET group.

We do agree that additional more robust and comprehensive studies need to be completed to understand the impact of gender minority only status on health of the gut microbiome. Thus, we have added text to the discussion to this point. **See lines 297-298:** Further, the SGM group did not include individuals identifying as lesbian nor did it include gender minority only individuals thus, findings do not apply to these minority groups.
